# Effect of Hyaluronic Acid-Containing Transfer Media (EmbryoGlue®) on the Live Birth Rate in Frozen Thawed Embryo Transfer Cycles

**DOI:** 10.7759/cureus.52713

**Published:** 2024-01-22

**Authors:** Nihar R Bhoi, Nitiz Murdia, Kshitiz Murdia, Vipin Chandra, Isha Suwalka, Walmik Mistari, Ritesh Aggrawal, Naval Shah, Dayanidhi Kumar

**Affiliations:** 1 Reproductive Medicine, Indira IVF Hospital Private Limited, Udaipur, IND; 2 Embryology, Indira IVF Hospital Private Limited, Udaipur, IND; 3 Clinical Research and Operations, Indira IVF Hospital Private Limited, Udaipur, IND; 4 Research and Publication, Indira IVF Hospital Private Limited, Udaipur, IND; 5 Reproductive Medicine, Indira IVF Hospital Private Limited, Patna, IND

**Keywords:** in vitro fertilization ivf, thawing, clinical pregnancy rate, live birth rate, embryoglue®

## Abstract

This retrospective cohort study examines the impact of EmbryoGlue® - a culture medium comprising high-concentration hyaluronan and low-concentration recombinant human albumin (rHA) - on assisted reproductive technology (ART) outcomes in 1,298 cycles across 13 centers. The study focused on live birth rates, clinical pregnancy, and miscarriage rates between a standard treatment arm and an EmbryoGlue® arm in frozen embryo transfer (FET) cycles. Propensity score matching ensured comparable baseline variables. Findings showed higher live birth rates (60.6% vs. 47.5%) and clinical pregnancies (69.5% vs. 57.6%) in the EmbryoGlue® group, correlating with factors like patient age and blastocyst transfer. Specifically, EmbryoGlue® showed a significant association with higher live birth rates (OR 1.593; CI 1.170-2.168; P = 0.003). These findings underscore the impact of personalized approaches and highlight EmbryoGlue®'s potential in improving successful embryo implantation, thus enhancing pregnancy rates in ART procedures. Univariate and multivariate analyses identified EmbryoGlue®, female age, and blastocyst transfer as predictors of live birth. EmbryoGlue® exhibited significance in improving clinical outcomes, mirroring previous studies' findings. Limitations in the study's design warrant further prospective research for validation. In conclusion, EmbryoGlue® appears promising for enhancing live birth rates in FET cycles, presenting a potential advancement in ART protocols.

## Introduction

Over the last decade, advancements in commercial culture media have resulted in better embryo development, quality, viability, and implantation [1]. Commercial media are formulated to provide the right balance of nutrients, hormones, and other factors needed for the growth and development of the embryo [2]. These media are designed to mimic the conditions found in the female reproductive tract and provide the necessary nutrients for embryo development. For sperm preparation, commercial media are used to wash and concentrate sperm, removing any impurities and debris that may be present in the semen. This process is essential to improving the chances of successful fertilization and embryonic development. For embryo transfer, commercial media are used to prepare the uterus and create an optimal environment for embryo implantation. These media contain hormones or other factors that help prepare the endometrium for implantation and support early embryo development [3].

Albumin is commonly used as a component of culture media in IVF procedures to provide a source of proteins and other macromolecules that are essential for embryo growth and development. It is typically added to culture media at a concentration of 0.1-0.5% and can help support embryonic development by providing a source of amino acids, fatty acids, and other nutrients [4]. Another important medium is hyaluronic acid (HA), a macromolecule belonging to the family of glycosaminoglycans (GAGs). It is a linear polymer composed of repeating disaccharide units of glucuronic acid and N-acetylglucosamine, and it is found naturally in the extracellular matrix of many tissues in the body [5]. HA can act as a biomimetic matrix, providing a supportive environment that allows embryos to attach and initiate the process of implantation. During the conventional embryo transfer procedure, embryos are typically cultured in a dish with a primary culture medium that provides the necessary nutrients for their growth [6]. However, when it comes to the time of transfer of the embryos into the uterus, the culture medium may not provide sufficient support for implantation. Adding hyaluronic acid to the culture medium makes the solution thicker and more viscous, mimicking the natural environment of the uterus. This can help the embryos adhere to the uterine lining and improve their chances of implantation [7].

EmbryoGlue® (VitroLife, Sweden) is typically composed of hyaluronan (high concentration of HA (0.5 mg/mL)), a natural substance in the female reproductive tract, and other nutrients (low concentration of recombinant human albumin (rHA = 2.5 mg/mL)) supporting the embryo. The embryo glue is believed to improve the chances of successful implantation by helping the embryo adhere to the endometrium and promoting communication between the embryo and the uterine environment [8].

However, some studies [9] have suggested that using EmbryoGlue® may improve the chances of successful embryo implantation, particularly in cases where the woman has experienced multiple failed IVF attempts or has other factors that may impact embryo implantation [10]. While some studies have suggested a positive effect on implantation rates, more research is needed to confirm these findings and determine the impact on the live birth rate. To accomplish this, we need to investigate whether the use of transfer media containing HA enhances the live birth rate in frozen embryo transfer (FET) cycles.

The main objective is to determine the impact of hyaluronan (hyaluronic acid)-containing transfer media, specifically EmbryoGlue®, on the outcomes of assisted reproductive technology (ART). The use of EmbryoGlue® is a relatively simple step in the IVF process. It involves placing the embryo in the culture medium for a short period before transferring it to the uterus. This simplicity makes it easy to incorporate into the existing IVF protocol.

## Materials and methods

Study design

This cohort (retrospective) multicenter study was carried out across 13 different locations of Indira IVF Hospital Private Limited, spanning from January 2015 to January 2022. All study protocols adhered to established operating standards. Data from a total of 1298 cycles were extracted from archived patient records at Indira IVF Hospital Private Limited in India, encompassing the timeframe from January 2015 to January 2022. The females aged 20-40 years, with a body mass index (BMI) of 18.0 to 35 kg/m2, embryo quality of 3/45 AA AB BA, and both self and donor cycles, were included. Any existing uncontrolled medical illness (diabetes, hypertension, thyroid disorder, congenital uterine anomaly, endometrial thickness <7 mm on the day of embryo transfer, ovarian cyst, and pelvic or systemic inflammatory disease) was excluded from the study. This study was designed as a two-arm study in which the standard treatment arm (group A) received conventional medium (n=649) and the second arm (group B) (n=649) received EmbryoGlue® for embryo transfer. In this study, the primary outcome measure was the live birth rate, and the secondary outcome measures were the clinical pregnancy rate and clinical miscarriage rate. Each case was followed till delivery either by patient visit or by telephonic follow-up (those who preferred different centres for pregnancy care). The loss of follow-up was excluded from the data. Approval of the study was obtained from the Indira IVF Hospital Private Limited Institutional Ethics Committee (ECR/1614/Inst/RJ/2021).

Study protocol

As per our standard protocol, all women were given standard treatment by antagonist protocol; the initial dose for stimulation was decided as per the age, AMH level, and AFC count, which were prepared from our patient data. The trigger planning was done once the major cohort of follicles reached 18-21 mm in size. After ovum pickup, the oocyte quality and an assessment of maturity status were done, and as a standard, all the oocytes were injected with single morphologically good sperm by Intracytoplasmic sperm injection (ICSI), cultured in single-step media, and assessed on D-5 for blastocyst.

Blastocyst grading and embryo transfer

Blastocysts were graded in accordance with the Gardner and Schoolcraft system of embryo grading and subsequently cryopreserved on either day 5 or day 6 of development. On the designated transfer day, careful assessments were carried out on the embryos to evaluate their viability, degree of expansion, and potential signs of degeneration. Viable day 5 (D5) or day 6 (D6) blastocysts exhibiting robust expansion were identified and chosen for the transfer process. The decision of whether to perform a single embryo transfer (SET) or a double embryo transfer (DET) was made based on individual patient characteristics and the clinical judgment of the consultant. Using a cook-soft catheter, the transfer procedure was performed under ultrasound (USG) guidance.

Endometrium preparation

For FET cycles, oestradiol valerate, in a dosage of 2 mg oral three times a day, was given for endometrial preparation in the subsequent hormone-free cycle following ovum pick-up. The administration of oestradiol valerate commenced on day 2 of either the natural or induced cycle. After 14 days of oestradiol treatment, the patient underwent an assessment of the endometrium's condition. For individuals exhibiting an endometrial thickness of 7 mm or more, levels of serum oestradiol (E2) and progesterone (P4) were assessed. In cases where P4 levels were below 1 ng/ml, progesterone supplementation was initiated. Participants with an endometrial thickness of less than 6 mm on the 14th day of hormone replacement therapy (HRT) were recommended for an increase in estrogen dosage, but they were subsequently excluded from the study. Those with an endometrial thickness ranging from 6 mm to 7 mm continued with the same dose and were re-evaluated after two to three days. Intramuscular administration of progesterone (100 mg) occurred once daily for a span of six days, leading up to the transfer of embryos at the blastocyst stage.

Thawing procedure

The institution follows a meticulously designed protocol using the Kitazato kit for thawing embryos, involving precise steps and specific solutions. This process includes thawing solution (TS) in two 4 mL vials, diluent solution (DS) in one 4 mL vial, and washing solution (WS) in another 4 mL vial. The process begins by warming the TS vial alongside a sealed Petri dish in a 37 °C incubator for over 1.5 hours, while the DS and WS are brought to room temperature between 25 and 27 °C. A cryovial with patient information is rapidly immersed into liquid nitrogen in a cooling rack and then labeled DS, WS1, and WS2 on a repro plate's lid.

The gentle inversion of DS and WS vials ensures proper mixing before dropping 300 μL of each solution into the corresponding wells on the repro plate. The plate is covered and placed on a microscope stage. The TS vial, along with the Petri dish, is taken out of the incubator, with its contents inverted twice for thorough mixing before being poured into the Petri dish. After carefully removing the cover straw from the cryovial, it is placed near the cooling rack's corner. With a Pasteur pipette and the cryovial still in liquid nitrogen, the countdown starts. The cryovial is swiftly immersed in TS on the microscope stage, and the embryo's position is adjusted using its black mark.

One minute later, the embryo is aspirated along with TS, positioned around 2 mm from the pipette's tip. Gradual displacement from TS to DS is facilitated by gently blowing out TS into the DS bottom, and the embryo is placed there for three minutes. This process is repeated in DS and WS1 to enable gradual displacement. Once in WS1, the embryo is aspirated with minimal WS1 using a Pasteur pipette, transferred to WS2's surface center, and allowed to descend to the bottom.

Subsequently, the embryo is moved to a culture dish with the appropriate medium and incubated at 37 °C for two hours to ensure complete recovery. For embryo glue use, an ideal incubation time of four to six hours is recommended. After thawing and post-thaw recovery, embryos can be placed in a culture dish with pre-incubated embryo glue, about 20-30 minutes before the planned embryo transfer. This method offers adequate time for the embryo to adapt to hyaluronan. The meticulous execution of these procedures ensures the optimal handling and preparation of embryos for transfer.

Embryo transfer and luteal phase support

ET was performed on the sixth day of P4 administration (after the completion of six dosages of injectable P4). Injectable progesterone was stopped after embryo transfer and luteal support was continued from the day of ET, as mentioned below.

The LPS consists of estradiol valerate (E2), 6 mg daily, until six weeks of GA. Progesterone 4 was supplemented in the form of vaginal gel (Crinone 8% gel) twice daily, and oral Dydrogesterone 10 mg twice daily, starting the day of ET. Other medications include folic acid, aspirin, and LMWH. This was continued until confirmation of the pregnancy. Further continuation depends on the status of the pregnancy.

Follow-up and assessment

A thorough assessment of pregnancy outcomes is essential in assisted reproductive techniques to monitor the success of embryo transfer procedures. In this study, a comprehensive approach was adopted to evaluate different aspects of pregnancy progression. The chemical pregnancy assessment at two weeks post-ET provided an early indication of pregnancy through the measurement of serum β-hCG levels. A threshold of 100 IU for serum β-hCG was chosen to establish the presence of biochemical pregnancy, taking into consideration the timing of blastocyst transfer and the Day 14 evaluation.

The confirmation of clinical pregnancy at four weeks post-ET was carried out through transvaginal ultrasound. This imaging technique allowed for the visualization of an intrauterine gestational sac and the fetal heartbeat, confirming the viability of the pregnancy. This step provided more definitive evidence of pregnancy progression and viability beyond the initial biochemical assessment.

The ongoing pregnancy assessment at 10 weeks post-ET, using transvaginal ultrasound, further solidified the understanding of the pregnancy's continuation. This evaluation at the 10-week mark, corresponding to 12 weeks of gestational age, allowed for a more comprehensive assessment of the pregnancy's viability and development.

Throughout these assessments, the consideration of concurrent medications and any adverse events was crucial. This ensured that any potential influences on pregnancy outcomes were accounted for and reviewed, contributing to the reliability of the study's findings. The structured schedule of assessments provided a consistent framework for the evaluation, enhancing the study's rigor and allowing for meaningful comparisons between different treatment arms.

Statistical analysis

Categorical variables were represented in terms of frequency (percentages), while continuous variables were expressed as mean ± standard deviations (SD). The normality of continuous data was evaluated using the Kolmogorov-Smirnov test. Subsequently, categorical variables were compared using the chi-square test or Fisher exact test as appropriate, whereas continuous variables were analyzed using the student t-test or Mann-Whitney U-test when applicable. To identify independent predictor variables for live birth, univariate, and multivariate logistic regression analyses were conducted. A significance level of p<0.05 was considered statistically significant for all analyses. The statistical analyses were executed using the Statistical Package for the Social Sciences (IBM SPSS), version 28.0.

We conducted a comparison between the standard treatment group and the EmbryoGlue® group within a propensity score-matched population. This approach was employed to mitigate any imbalances and establish comparability in baseline variables between the two groups. The matching ratio between the standard treatment group and the EmbryoGlue® group was maintained at 1:1. Propensity scores were computed using binary logistic regression along with the nearest neighborhood technique, based on the following baseline variables: Wife Age, BMI, AMH, and Number of embryos transferred.

## Results

**Figure 1:**
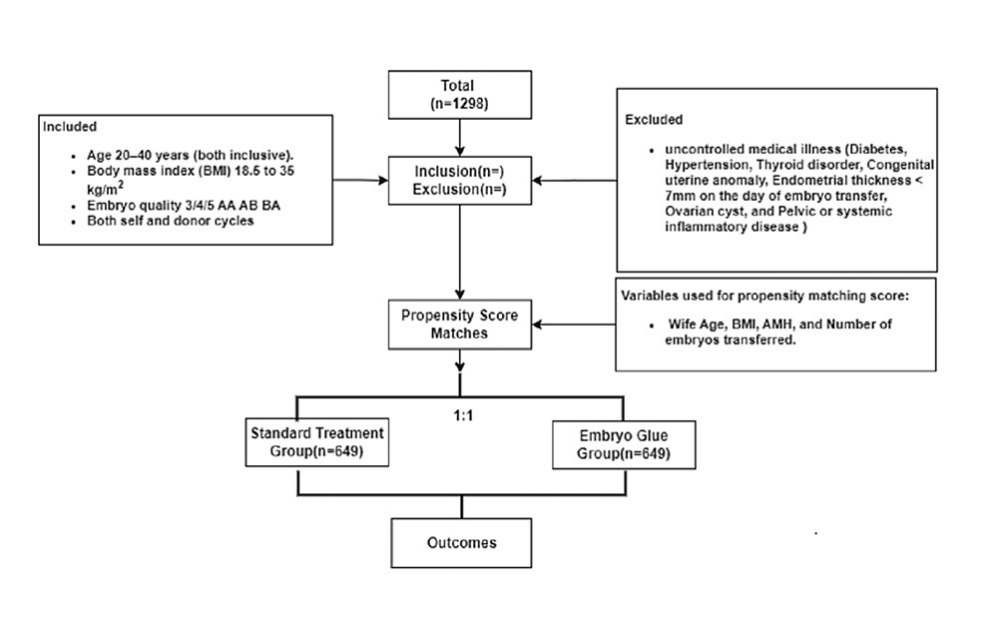
Flowchart for sample selection and final study population.

This retrospective study involved a thorough analysis of a total of 1,298 cycles, with 649 cycles allocated to each of the two arms: the standard treatment arm (group A) and the EmbryoGlue® arm (group B). To ensure a balanced comparison of baseline variables, propensity score matching was meticulously employed. Following the implementation of propensity score matching, the final analysis incorporated 649 cycles from the standard treatment group and an equivalent number of cycles (649) from the EmbryoGlue® group (Figure 1).

Following this matching process, the average age of wives in group A was 31.93±4.24 years, while in group B, it was 31.84±4.16 years, and this difference was not found to be statistically significant (p=0.687). Table 1 defines the outcome definitions with a detailed procedure for calculation. Furthermore, crucial baseline factors, including BMI, anti-Mullerian hormone (AMH) levels, and the number of embryos transferred, were also harmoniously matched across both groups, indicating their comparable nature (Table 2).

**Table 1:** Outcome measures.

Outcome	Definition	Calculation
Live birth rate	This metric gauges the number of successful deliveries with at least one live birth for every 100 cycles of embryo transfer.	Live birth rate = (number of live births/number of embryo transferred cycles) × 100
Clinical pregnancy rate	This rate signifies the detection of a fetal heartbeat using transvaginal ultrasound during the 6th week of gestation.	Clinical pregnancy rate = (number of diagnosed pregnancies with gestational sacs at 6th-week number of embryo transferred cycles) × 100.
Clinical miscarriage rate	This rate reflects the number of spontaneous pregnancy losses where a gestational sac or sacs were previously observed before the 20th week of gestation. Clinical miscarriage rate = (total number of miscarriage cases before 20th week/number of clinical pregnancy cases) × 100.	Clinical miscarriage rate = (total number of miscarriage cases before 20th week/number of clinical pregnancy cases) × 100.
Biochemical loss rate	This rate evaluates early pregnancy losses occurring within five weeks after implantation in relation to the number of confirmed biochemical pregnancies.	Biochemical loss rate = (number of early pregnancy losses/number of confirmed biochemical pregnancies) × 100.
Ongoing pregnancy rate	This rate is based on the presence of gestational sacs with a heartbeat at the 12th week of gestation.	Ongoing pregnancy rate = (number of gestational sacs with heartbeat at 12th week/number of embryo transferred cycles) × 100.
Implantation rate	This rate quantifies the number of gestational sacs observed through transvaginal ultrasound at the 6th week of gestation in relation to the number of embryos transferred.	Implantation rate = (number of gestational sacs observed at 6th week/total number of embryos transferred) × 100
MII rate	This rate represents the maturity of oocytes by comparing the number of matured oocytes to the number of retrieved oocytes.	MII rate = (number of matured oocytes/number of retrieved oocytes) × 100
Retrieval rate	This rate assesses the efficiency of oocyte retrieval by calculating the number of retrieved oocytes per 100 expected oocytes from follicles larger than 14 mm in diameter on the trigger day.	The formula for the retrieval rate is: retrieval rate = (number of retrieved oocytes/number of expected oocytes) × 100.
Blast rate	This rate indicates the proportion of successfully formed blastocysts in relation to the total number of matured oocytes injected.	Blast rate = (total number of blastocysts formed/total number of MII injected) × 100.
Good blast rate	This rate evaluates the quality of formed blastocysts (grade-1, indicated as AA, AB, BA) by comparing them to the total number of matured oocytes injected.	Good blast rate = (total number of grade-1 blastocysts/total number of MII injected) × 100.

**Table 2:** Baseline demographic features. ^!^Mann–Whitney, ^#^Independent t-test.

Characteristics	Standard treatment (n=649)	EmbryoGlue® (n=649)	P-value
Female age at FET, years (mean ± SD)	31.93±4.24	31.84±4.16	0.687^#^
Male age, years (mean ± SD)	35.43±5.02	35.76±4.69	0.218^#^
Female body mass index (kg/m^2^) (mean ± SD)	25.37±4.13	25.36±4.22	0.972^#^
AMH (mean ± SD)	3.52±3.01	3.31±2.78	0.142^!^
Stimulation protocol, n (%)			<0.001
Antagonist	590/649(90.9%)	389/400(97.3%)	
Minimal	59/649(9.1%)	11/400(2.8%)	
Sperm washing technique, n (%)			<0.001
Density gradient	41/611(6.7%)	8/612(1.3%)	
Qualis	49/611(8%)	12/612(2%)	
Swim-up	521/611(85.3%)	592/612(96.7%)	
Male factor, n (%)	454(70.0%)	422(65%)	0.058
Number of previous IVF attempts, n (%)			<0.001
0	585(90.1%)	506(78.0%)	
1	57(8.8%)	119(18.3%)	
2	7(1.1%)	24(3.7%)	
Number of patients with PGT-A, n (%)	15(2.3%)	22(3.4%)	0.243

The average MII (matured oocytes) rate and expected oocytes were observed to be statistically significant. Group B (EmbryoGlue® arm) had a higher MII rate (73.64±18.49) compared to group A (standard treatment arm) (68.76±16.72) (p<0.001), indicating better oocyte maturity with the use of EmbryoGlue®. Additionally, the expected oocytes were higher in group B (14.28±8.23) compared to group A (13.02±8.04) (p=0.013). There was no significant difference between the two groups in terms of the number of patients with PGT-A testing. However, the number of previous IVF attempts was significantly different between the two groups, with a higher percentage of patients in the standard treatment group (group A) having no previous IVF attempts (Table 2).

Table 2 provides data on factors related to embryo transfer in both treatment groups. The mean endometrial thickness at the start of progesterone administration was slightly higher in the EmbryoGlue® group (group B) compared to the standard treatment group (group A), although the difference was not statistically significant. The duration of progesterone administration was similar between the two groups. There was no significant difference in the number of blastocysts transferred between the groups, with the majority of patients in both groups receiving two blastocysts. For SET, the proportion of blastocysts with excellent or good morphology was higher in the EmbryoGlue® group compared to the standard treatment group, but the difference was not statistically significant. For DET, the proportion of blastocysts with excellent or good morphology was similar between the two groups (Table 3).

**Table 3:** Comparison of treatment outcomes and parameters in IVF/ICSI cycles using standard treatment vs. EmbryoGlue®. ^#^Independent t-test, morphology grading: excellent (3AA,4AA,5AA), good (3,4,5,6 AB or BA), average (3,4,5,6 BB or AC or CA), poor (3,4,5,6 BC or CC).

Variables	Standard treatment (n=649)	EmbryoGlue® (n=649)	P-value
Endometrial thickness at the day of P start, mm (mean ± SD)	8.94±1.24	9.05±1.00	0.079^#^
Duration of progesterone administration, n (%)			0.808
Day 6	612(94.3%)	614(94.6%)	
Day 7	37(5.7%)	35(5.4%)	
Number of blastocysts transferred n (%)			0.298
1	97(14.9%)	84(12.9%)	
2	552(85.1%)	565(87.1%)	
Blastocyst morphology of SET, n (%)			0.071
Excellent + good	89(91.8%)	83(98.8%)	
Average	3(3.1%)	1(1.2%)	
Poor	5(5.2%)	0(0%)	
Blastocyst morphology of DET (first embryo), n (%)			0.179
Excellent + good	529(95.8%)	549(97.2%)	
Average	20(3.6%)	16(2.8%)	
Poor	3(0.5%)	0(0%)	
Blastocyst morphology of DET (second embryo), n (%)			0.200
Excellent + good	487(88.2%)	507(89.7%)	
Average	56(10.1%)	55(9.7%)	
Poor	9(1.6%)	3(0.5%)	
βHCG rate, n/n (%)	406/649(62.6%)	494/649(76.1%)	<0.001
Biochemical loss rate, n/n (%)	32/406(7.9%)	43/494(8.7%)	0.657
Clinical pregnancy rate, n/n (%)	373/649(57.6%)	451/649(69.5%)	<0.001
Clinical miscarriage rate, n/n (%)	65/373(17.4%)	58/451(12.9%)	0.067
Live birth rate, n/n (%)	308/649(47.5%)	393/649(60.6%)	<0.001
Multiple live birth rate, n/n (%)	100/649(15.4%)	132/649(20.3%)	0.021

Moving on to reproductive outcomes, the EmbryoGlue® arm (group B) demonstrated significantly higher rates of biochemical pregnancy, clinical pregnancy, live birth, and multiple live births compared to the standard treatment arm (group A) (Table 3). The biochemical pregnancy rate was 76.1% in group B and 62.6% in group A (p<0.001). The clinical pregnancy rate was 69.5% in group B and 57.6% in group A (p<0.001). The live birth rate was 60.6% in group B and 47.5% in group A (p<0.001). The multiple live birth rate was 20.3% in group B and 15.4% in group A (p=0.021).

Univariate logistic regression analysis identified several independent factors significantly associated with predicting live births. These factors include female age, the number of blastocysts transferred (DET vs. SET), patients with PGT-A, duration of progesterone administration (6th day vs. 7th day), endometrial thickness on the day of progesterone start, and the use of EmbryoGlue® (Table 4).

**Table 4:** Univariate and multivariate analysis to find out independent predictors of live birth.

Characteristics	Univariate analysis	P-value	Multivariate analysis	P-value
	Odds ratio (95% CI)		Odds ratio (95% CI)	
Female age at FET, years	0.933(0.908–0.958)	<0.001	0.927(0.893–0.963)	<0.001
Female body mass index (kg/m^2^)	0.969(0.943–0.997)	0.028	0.976(0.942–1.011)	0.175
Patients with PGT-A	0.306(0.147–0.636)	0.002	0.416(0.158–1.100)	0.077
Number of blastocysts transferred (DET) (ref. SET)	2.173(1.573–3.003)	<0.001	1.618(1.086–2.410)	0.018
Duration of progesterone administration (6th day) (ref. 7th day)	1.693(1.045–2.740)	0.032	1.575(0.881–2.814)	0.125
Endometrial thickness at the day of P start, mm	1.167(1.056–1.290)	0.002	1.147(1.014–1.298)	0.029
EmbryoGlue® (ref. standard treatment)	1.700(1.364–2.118)	<0.001	1.593(1.170–2.168)	0.003

Furthermore, a multivariate logistic regression analysis was performed to assess the independent factors predicting live births. The results showed that female age, the number of blastocysts transferred (DET vs. SET), endometrial thickness on the day of progesterone start, and the use of EmbryoGlue® remained significant independent factors associated with predicting live birth (Table 4).

The results of the study compared standard treatment and embryo glue treatment (EG) for two age groups (<35 years and ≥35 years). For patients aged <35 years, the clinical pregnancy rate was 62.3% in the standard treatment group and significantly higher at 71.7% in the embryo glue treatment group (p-value = 0.002). The live birth rate was 51.9% in the standard treatment group and considerably higher at 63.4% in the embryo glue treatment group (p-value < 0.001). For patients aged ≥35 years, the clinical pregnancy rate was 44.6% in the standard treatment group and notably higher at 63.6% in the embryo glue treatment group (p-value < 0.001). The live birth rate was 35.6% in the standard treatment group and notably higher at 52.8% in the embryo glue treatment group (p-value = 0.001).

In conclusion, the retrospective study comparing standard treatment to the addition of EmbryoGlue® in FET cycles revealed that the use of EmbryoGlue® was associated with significantly higher rates of MII oocytes, expected oocytes, biochemical pregnancy, clinical pregnancy, live birth, and multiple live births. The use of EmbryoGlue® along with specific patient and embryo characteristics, such as female age and the number of blastocysts transferred, were identified as significant independent predictors of live birth. These findings suggest that EmbryoGlue® may be a valuable option to enhance reproductive outcomes in FET cycles.

## Discussion

Over the years, various modifications have been made to the embryo transfer medium in order to improve live birth and clinical pregnancy rates [9]. Our study focuses on the modification that involves the use of protein supplementation, with albumin being the most commonly used protein in embryo transfer mediums [10]. It also acts as a lubricant, facilitating easy embryo handling and preventing embryo adherence to the culture dish. In this study, the embryos were cultured with 10 mg/mL of rHA and a lower concentration of HA in G-2™, Vitrolife, and compared to the EmbryoGlue® medium.

The objective of the present study was achieved and illustrates that the use of EmbryoGlue® significantly improved the clinical pregnancy rate compared to the standard treatment group [11]. This finding is consistent with previous studies by Schoolcraft et al. [12] and Balaban et al. [13], which have also reported higher clinical pregnancy rates associated with the use of EmbryoGlue®. Additionally, Adeniyi et al. [10] found that EmbryoGlue® enhanced clinical pregnancy and implantation rates in both fresh and frozen-thawed embryo transfer cycles [14]. The authors suggested that the high viscosity of EmbryoGlue® may physically protect embryos treated with assisted hatching during frozen-thawed cycles.

The mechanism of action of hyaluronan in promoting embryo implantation may be attributed to several factors. Hyaluronan can bind to specific cell surface receptors, creating a favorable environment for embryo attachment and implantation. It also absorbs and retains water, leading to increased hydration and lubrication of tissues, which may aid in creating a conducive environment for embryo implantation by providing physical support, reducing friction, and facilitating embryo movement through the reproductive tract [15].

The live birth rate, a crucial measure of success in fertility treatments, was significantly higher in the EmbryoGlue® group compared to the standard treatment group in this study. This outcome aligns with the ultimate goal of achieving a healthy pregnancy resulting in the birth of a healthy baby, as previously highlighted by Wang et al. The live birth rate reflects the percentage of embryo transfers that resulted in at least one live birth, making it a clinically meaningful measure of success in assisted reproductive treatments.

Other studies, such as the one conducted by Adeniyi et al., have also reported positive outcomes with the use of hyaluronan-enriched embryo transfer medium in ICSI cycles. While the study found a significantly higher pregnancy rate in the group that used the hyaluronan-enriched medium, there were no significant differences in implantation rate, miscarriage rate, or live birth rate. These findings suggest that hyaluronan may primarily influence early pregnancy outcomes, such as pregnancy rates [16].

A study conducted by Fadhil et al. further supports the notion that EmbryoGlue® may improve pregnancy rates, particularly in women aged 35 and above [17]. The study found significantly higher pregnancy rates in subgroup AII (women aged 35 and above) who received EmbryoGlue® compared to subgroup BII, which received a conventional medium. In our study for patients aged <35 years, the clinical pregnancy rate was 62.3% in the standard treatment group and significantly higher at 71.7% in the embryo glue treatment group (p-value = 0.002).

It is essential to acknowledge that miscarriage rates were comparable between the EmbryoGlue® group and the standard treatment group in this study. Miscarriage rates can be influenced by various factors, such as maternal age, underlying medical conditions, and embryonic chromosomal abnormalities. As a result, these factors may have contributed to the lack of significant differences in miscarriage rates between the two groups.

Overall, the results of this study suggest that the use of EmbryoGlue® in IVF treatments may lead to significant improvements in biochemical pregnancy rate, clinical pregnancy rate, live birth rate, and multiple live birth rate compared to the standard treatment group. However, it is essential to consider the potential risks and benefits of using EmbryoGlue®, particularly the increased risk of multiple pregnancies.

As with any retrospective study, this study has certain limitations, which might introduce biases and hinder the establishment of causal relationships between the identified factors and live birth outcomes. Considering the retrospective nature, there was missing data from previous ART outcomes, so we could not analysed the same. Future prospective randomized controlled trials may be necessary to further validate and confirm these findings. Considering the retrospective nature, there was missing data from previous ART outcomes, so we couldn’t analysed the same. Nevertheless, this study contributes valuable information on the potential benefits of using EmbryoGlue® in frozen embryo transfer cycles, providing a basis for further research and clinical decision-making in assisted reproductive treatments.

## Conclusions

It can be concluded that the use of EmbryoGlue® as an embryo transfer medium can result in significantly higher rates of clinical pregnancy, live birth, and multiple live births compared to conventional culture media. The study also found that EmbryoGlue® did not significantly impact endometrial thickness, progesterone administration duration, or blastocyst morphology.
